# Glucose variability during delirium in diabetic and non-diabetic intensive care unit patients: A prospective cohort study

**DOI:** 10.1371/journal.pone.0205637

**Published:** 2018-11-15

**Authors:** Kris van Keulen, Wilma Knol, Svetlana V. Belitser, Irene J. Zaal, Paul D. van der Linden, Eibert R. Heerdink, Toine C. G. Egberts, Arjen J. C. Slooter

**Affiliations:** 1 Department of Clinical Pharmacy, Tergooi Hospitals, Hilversum, the Netherlands; 2 Division of Pharmacoepidemiology and Clinical Pharmacology, Utrecht University, Utrecht, the Netherlands; 3 Department of Geriatric Medicine and Expertise Centre Pharmacotherapy in Old Persons (EPHOR), University Medical Centre Utrecht, Utrecht, the Netherlands; 4 Department of Intensive Care Medicine, Brain Centre Rudolf Magnus, University Medical Centre Utrecht, Utrecht, the Netherlands; 5 Department of Clinical Pharmacy, University Medical Centre Utrecht, Utrecht, the Netherlands; Florida International University Herbert Wertheim College of Medicine, UNITED STATES

## Abstract

**Purpose:**

To determine whether glucose variability is altered during delirium days compared to non-delirious days in critically ill patients with and without diabetes in the intensive care unit (ICU).

**Materials and methods:**

Critically ill patients with delirious and non-delirious days during ICU stay were included from a prospective cohort study which was conducted from January 2011- June 2013. Glucose variability was measured each observation day using various definitions (change in mean glucose, standard deviation, mean absolute glucose, daily delta and occurrence of hypo- and hyperglycemia). Mixed-effects models and generalized mixed-effects models with logit link function were performed to study the association between delirium and glucose variability, adjusting for potential confounders.

**Results:**

With the exception of the risk of hypoglycemia, delirium was not linked to higher glucose variability using the various definitions of this estimate. For hypoglycemia, we did find an association with delirium in diabetic patients (OR adj.: 2.78; 95% CI: 1.71–6.32, p = 0.005), but not in non-diabetic patients (OR adj.: 1.16; 95% CI: 0.58–2.28, p = 0.689).

**Conclusions:**

Despite the positive association between delirium and hypoglycemia in critically ill patients with diabetes, delirium was not associated with more pronounced glucose variability. Our findings suggest that glucose levels should be monitored more closely in diabetic patients during delirium at the ICU to prevent hypoglycemia.

## Introduction

Delirium is a frequently observed complication in patients in an intensive care unit (ICU) [1–3], that has been associated with long-term cognitive impairment [4,5], prolonged length of ICU stay [6] and with increased health care costs [6,7]. The pathophysiology of delirium is complex and heterogeneous. Metabolic disorders such as hypo- and hyperglycemia have been identified as risk factors for delirium onset, but extensive research is lacking [8–11]. To improve patient related outcomes, identification of modifiable factors in delirium need to be further explored.

Tight glucose control has been implemented as regular care in critically ill patients to reduce extreme glucose deviations as hypo- and hyperglycemia, glucose variability and to decrease the mean glucose concentration with decreased mortality risk as result [12,13]. However, the optimal blood glucose range in tight glucose control is controversial. Intensive glucose control (glucose target between 4.5–6.0 mmol/l (81.0–108.1 mg/dL)) has been shown to increase mortality rates compared to conventional glucose control (glucose target ≤ 10.0 mmol/l (180.1 mg/dL)) [14]. The occurrence of hypoglycemia during intensive glucose control may be responsible for this increased risk of death [15]. Furthermore, it has been reported that the mortality rate after hyperglycemia is higher in non-diabetic patients compared to diabetic patients [16,17], due to adaptive mechanisms to chronic hyperglycemia in patients with diabetes [18].

Glucose variability has been associated with higher mortality risk in critically ill patients [19,20]. A gold standard for measurement of blood glucose fluctuations is lacking [21–23]. Glucose fluctuations were frequently reported as glucose variability and refer for example to mean glucose concentration, mean absolute glucose (MAG) change, standard deviation (SD) or hypo- and hyperglycemia.

Delirium and glucose variability have both been associated with negative outcomes, but their mutual relation has been poorly studied. Higher glucose values have been reported in critically ill patients with hyperactive delirium compared to critically ill patients with non-hyperactive delirium [10]. Given that delirium results from acute illness, it is plausible that this acute illness may increase activity of hypothalamic-pituitary-adrenal axis leading to increased cortisol release and subsequent decreased peripheral insulin sensitivity, thereby contributing to hyperglycemia. It is unclear whether glucose variability is higher during delirium within the window of glucose control during ICU admission.

The aim of this study was to determine whether estimates of glucose variability are altered during delirium in critically ill patients with and without diabetes in the ICU.

## Materials and methods

### Setting, study design and population

Data were used from a prospective cohort study conducted in the 32-bed mixed ICU of the University Medical Centre Utrecht (UMCU), the Netherlands. All patients hospitalized for longer than 24 hours on the ICU in the period from January 2011 to June 2013 were included in this study, except in the case of neurological illness, if delirium assessment was impossible or patients were unable to speak Dutch or English. The local Institutional Review Board waived the need for informed consent in this non-interventional investigation (IRB 010/056/c and 12/421/c) and approved further research with the anonymous data.

The mental status of all ICU patients was daily classified by the research team as ‘delirious’, ‘awake and non-delirious’ or ‘comatose’ using a 5-step validated algorithm (interobserver agreement, 0.94–0.97; sensitivity, 0.75; and specificity, 0.85) [24]. This multistep algorithm incorporates a review by a research nurse of all Confusion Assessment Method for the ICU (CAM-ICU) [25] assessments conducted by the bedside nurses, whether delirium treatment was initiated and a meticulous chart review for the presence of documented terms clinically associated with delirium. When delirium could not be ruled in or out using this procedure, the research nurse conducted an additional CAM-ICU assessment. Delirium episodes were recorded and delirium subtype was classified using the 3 hourly registered RASS scores (10 point scale ranging -5 (comatose) to +4 (heavily agitated)) [26]. A delirium episode ended if a patient had a classification of ‘awake and non-delirious’ or a classification of ‘comatose’ for at least two days.

For this study, patients with delirious and non-delirious observation days were selected from the study cohort. In case of one delirious episode during ICU stay all observation days were included until ICU discharge. In case of more than one delirious episode, observation days until the start day of the second delirious episode were included for that patient. Patients were excluded if there was no glucose value available during a delirious episode or during a non-delirious episode. Observation days were excluded from the study if there were no glucose values available or if the observation day was classified as ‘comatose’.

### Data collection

Trained, assigned physicians collected data (baseline and per day) from all ICU patients including demographic data, (chronic) co-morbidities and medication use, ICU admission characteristics, daily physiological measurements and vital signs, and therapeutic interventions. Diabetes was marked present if noted in the medical record or if patients used insulin and/ or oral antidiabetic drugs at ICU admission. Current alcohol intake was marked as positive if patients used more than three units of alcohol per day, as documented in the medical records or history. Current smoking was marked as positive if smoking was written in the medical records or history. Planned admissions were those admissions which could be postponed for at least 12 hours without adverse consequences. The Acute Physiology and Chronic Health Evaluation (APACHE) IV classification was used to determine the admission diagnosis, severity of disease, and infection at ICU admission [27]. The extent of chronic comorbidities were measured with Charlson Comorbidity Index (CCI) [28]. The Sequential Organ Failure Assessment (SOFA) score without central nervous system component was used daily to classify severity of disease [29]. The presence of severe sepsis or septic shock was classified using international sepsis definitions at the time of study [30–33].

During the study period, a glucose regulation protocol was used to maintain the target glucose concentration during ICU admission between 5.0 and 8.0 mmol/l (90.1–144.1 mg/dL) (S1 Table), except in those ICU-patients with a low risk of prolonged hyperglycemia such as per- and postoperative patients with one bolus injection of dexamethasone. Continuous insulin infusion was initiated in patients with diabetes and in ICU-patients with a (drug-induced) glucose concentration > 8.0 mmol/l. Glucose levels were measured on fixed time points between 0.5–4 hours after the last glucose measurement (details are described in the glucose regulation protocol S1 Table) from blood samples obtained from an arterial catheter using BeckmanCoulter AU5800 (Beckman Coulter Inc., Brea CA, USA) or if arterial catheter was absent by finger stick using Precision Xceed Pro (Abbott, Abbott Park, USA). Glucose levels were automatically stored in the electronic patient data management system (EPDMS, MetaVision, version 5.45, iMDsoft).

Medication use (drug, dose, route and time of administration including total parenteral nutrition) and glucose measurements (concentration and time of measurement) were retrieved from the EPDMS and added to the prospectively collected data. Continuous infusions, such as insulin, were recorded in the EPDMS, including end date and time of administration. A change in infusion rate resulted in a new medication record. If a continuous infusion covered more than one day, the dose per day was calculated using the ratio between infusion times of both days. Energy intake was defined as the sum of daily caloric intake from continuous infusion of glucose, total parenteral or enteral nutrition, and high caloric medication, such as propofol.

### Outcome

The primary outcome was the within-patient difference in glucose variability during delirious and non-delirious observation days. Glucose variability was measured each observation day, expressed by the following five measures:

1. mean glucose concentration (mmol/l)
2. SD of all glucose levels (mmol/l)
3. MAG change, defined as the mean absolute glucose change per hour (mmol/l/hour). To calculate the MAG, all absolute changes in blood glucose levels were added up and were divided by the time between first and last glucose levels (in hours) [19].
4. Daily delta, defined as the difference of daily maximum and daily minimum glucose concentration (mmol/l)
5. Hypo- and hyperglycemia. Hypoglycemia was defined as a glucose concentration <3.5 mmol/l (63.1 mg/dL) and severe hypoglycemia was defined as a glucose concentration <2.2 mmol/l (39.6 mg/dL). Hyperglycemia was defined as glucose concentration > 8.0 mmol/l (144.1 mg/dL) and severe hyperglycemia as glucose concentration > 11.0 mmol/l (198.2 mg/dL).

### Data analyses

Patient and observation day characteristics were reported as numbers with percentages in the case of nominal data and means with SD or median with interquartile range (IQR) in the case of continuous data. Continuous data were compared using Student independent sample t tests when the data was normally distributed; otherwise the Mann-Whitney U test was used. Chi-square tests were used to compare nominal data.

Characteristics of delirious and non-delirious days in non-diabetic and diabetic patients were compared in a multilevel technique using linear mixed-effects models for continuous characteristics and generalized mixed-effects models with logit link function for dichotomous characteristics. Statistical significance was considered at p-value <0.05, when appropriate 95% bootstrap percentile confidence intervals (CIs) were expressed. Two-stage bootstrap resampling procedure with ‘patient’ as cluster variable was used for obtaining CI’s and p-values from 1000 replications.

In the case of one glucose concentration per day the mean glucose concentration, SD and the difference of daily maximum and minimum could not be calculated. The MAG change was calculated if there were more than two glucose levels per day available. Hyperglycemia and hypoglycemia were described as dichotomous outcome per observation day, but glucose values were analysed individually. Linear mixed-effects models and generalized mixed-effects models with logit link function were used as multilevel techniques to test whether delirium was associated with increased glucose variability. The effects were expressed as regression coefficients or odds ratios, both with bootstrap 95% CIs. Covariates were included in the model as fixed effects, when possible as time dependent covariate. The use of medication was classified dichotomous per day. All models included random effects for ‘patient’.

The degree of glucose variability depends on diabetic status, [18] therefore separate models were developed for patients without and with diabetes. The adjusted models always included the following covariates; age, gender, total dose of insulin (bolus injection and continuous infusion) in the 30 minutes before glucose measurement or total dose of insulin per day and energy infusion in the 30 minutes before glucose measurement or energy infusion per day. Confounders were selected based on p-values (< 0.05) and effect sizes. The following variables were tested as potential confounders: age, gender, body mass index (BMI), current alcohol intake, current smoking, admission type, planned admission, confirmed infection, APACHE IV-score, CCI, SOFA-scores, support of mechanical ventilation, presence of severe sepsis or septic shock, number of observation day, length of stay (LOS) at ICU, the use of antipsychotic drugs, norepinephrine, corticosteroids, clonidine, ACE-inhibitors, cyclosporine or tacrolimus, beta-blockers and beta-agonists. All statistical analyses were carried out with R version 3.2.3 with package ‘lme4’ (R Foundation for Statistical Computing, Vienna, Austria).

## Results

**Fig 1:**
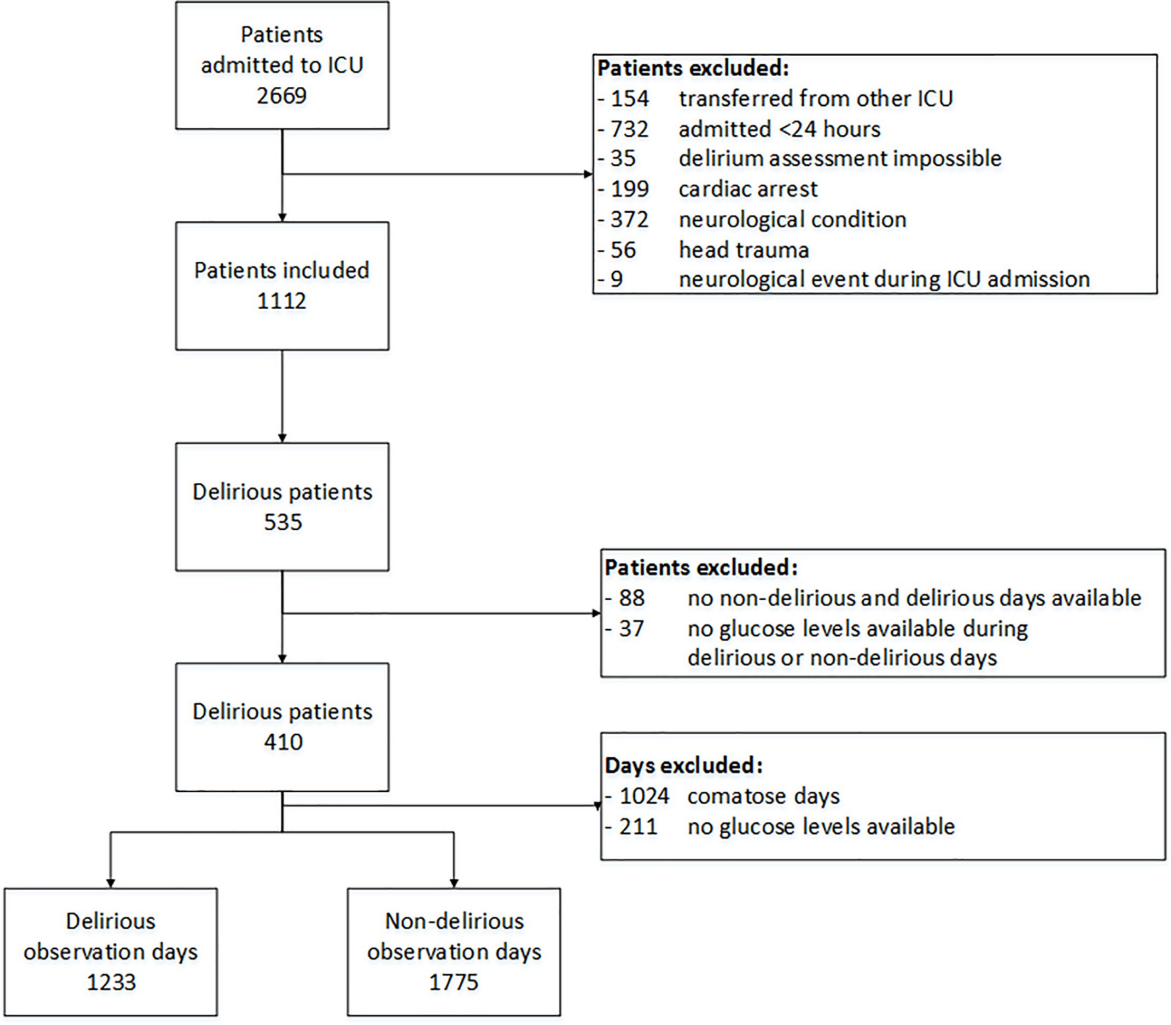
Flowchart of the study population. ICU = intensive care uni.

During the study period, 2669 patients were admitted to the ICU and of whom 1557 patients were excluded. Delirium was diagnosed in 535 patients. Of those patients, 125 patients were excluded: 88 (16.4%) patients because they had only delirious or comatose observation days during their ICU admission and 37 (6.9%) patients because of the absence of glucose values during delirious or non-delirious observation days. Therefore, the final population consisted of 410 patients with 1233 delirious and 1775 non-delirious observation days (Fig 1).

Patient characteristics are shown in Table 1. Diabetic patients were on average older, had a higher BMI and had a higher APACHE-IV score compared to non-diabetic patients. The number of delirious days was higher in diabetic patients compared to non-diabetic patients. Diabetic patients had a higher maximum glucose concentration in the first twenty-four hours of ICU-stay than patients without diabetes.

**Table 1:** Patient characteristics of the study population.

Characteristic	ICU patients (n = 410)		
	Non-diabetic patients* (n = 323)	Diabetic patients* (n = 87)	P- value
Age, mean (SD)	61.7 (14.6)	66.7 (12.0)	0.001^a^
Male gender, n (%)	203 (62.8)	54 (62.1)	0.894^b^
BMI in kg/m^2^, mean (SD)	25.3 (5.3)	29.0 (8.7)	< .001^a^
Current alcohol intake ^†^, n (%)	19 (5.9)	4 (4.6)	0.644^b^
Current smoking^†^, n (%)	31 (9.6)	4 (4.6)	0.139^b^
Diagnose*, n (%)			0.494^b^
Medical	133 (41.2)	42 (48.3)	
Surgery elective	97 (30.0)	23 (26.4)	
Surgery emergency	93 (28.8)	22 (25.3)	
Planned admission*, n (%)	91 (28.2)	22 (25.3)	0.593^b^
Confirmed infection*, n (%)	115 (35.6)	38 (43.7)	0.167^b^
Diabetes mellitus and organ damage*, n (%)	N.A.	7 (8.0)	N.A.
APACHE IV- score*, mean (SD)	77.5 (24.9)	88.1 (28.1)	0.001^a^
CCI^†^, mean (SD)	7.4 (6.5)	8.7 (6.2)	0.099^a^
Delirium days first episode, mean (SD)	3.1 (3.7)	4.2 (5.4)	0.085^a^
Subtype delirium, n (%)			0.384^b^
Hypoactive	101 (31.3)	23 (26.4)	
Hyperactive	0 (0.0)	0 (0.0)	
Mixed type	222 (68.7)	64 (73.6)	
One day episode, n (%)	142 (44.0)	29 (33.3)	0.074^b^
> 1 delirium episode, n (%)	92 (28.5)	33 (37.9)	0.089^b^
Number of delirious days, median (IQR)	2 (1–3)	2 (1–5)	0.019^c^
Number of non-delirious days, median (IQR)	3 (2–5)	3 (2–5)	0.686^c^
Max. glucose concentration in first 24h in mmol/l, mean (SD)	10.0 (2.7)	12.6 (4.0)	< .001^a^
ICU LOS in days, median (IQR)	9 (5–20)	10 (5–21)	0.425^c^
ICU mortality, n (%)	39 (12.1)	12 (13.8)	0.666^b^

SD = standard deviation, BMI = Body mass index, APACHE = Acute Physiology and Chronic Health Evaluation, CCI = Charlson comorbidity index, ICU = intensive care unit, LOS = Length of stay, DM = diabetes mellitus, n.a. = not applicable

† at hospital admission

* at ICU admission

^a^ Student independent sample t test

^b^ Chi-square test

c Mann-Whitney U test

Table 2 shows the characteristics of delirious and non-delirious days in non-diabetic and diabetic patients. During delirious days, diabetic and non-diabetic patients had more often insulin infusions, had more insulin rate adjustments, and had a higher average of numbers of glucose measurements in comparison with non-delirious days.

**Table 2:** Variables per observation day in critically ill patients during their stay at the intensive care unit.

Variables	Non-diabetic patients (n = 323)			Diabetic patients (n = 87)		
	Delirious days (n = 908)	Non- delirious days (n = 1395)	P- value	Delirious days (n = 325)	Non- delirious days (n = 380)	P- value
**Illness**						
Mean SOFA score, mean (SD)	6.0 (3.1)	4.7 (3.0)	< .001^a^	6.2 (3.1)	4.8 (2.8)	0.072^a^
Mechanical ventilation, n (%)	769 (84.7)	1045 (74.9)	< .001^b^	253 (77.8)	285 (75.0)	0.029^b^
Severe sepsis or septic shock, n (%)	280 (30.8)	223 (16.0)	< .001^b^	119 (36.6)	93 (24.5)	0.272^b^
Max Richmond Agitation Sedation Score, n (%)			< .001^a^			< .001^a^
Deep coma (-5 or -4)	1 (0.1)	4 (0.2)		0 (0.0)	0 (0.0)	
Light sedated (-3, -2, -1)	180 (19.8)	178 (12.8)		50 (15.4)	46 (12.1)	
Alert, Calm (0)	166 (18.3)	844 (60.5)		55 (16.9)	247 (65.0)	
Agitated (+1,+2,+3,+4)	561 (61.8)	369 (26.5)		220 (67.7)	87 (22.9)	
**Medication**						
Antipsychotics, n (%)	514 (56.6)	201 (14.4)	< .001^b^	205 (63.1)	67 (17.6)	< .001^b^
Oxazepam, n (%)	137 (15.1)	312 (22.4)	0.373^b^	53 (16.3)	45 (11.8)	0.100^b^
Clonidine, n (%)	191 (21.0)	137 (9.8)	0.039^b^	82 (25.2)	32 (8.4)	0.001^b^
Norepinephrine, n (%)	342(37.7)	347(24.9)	< .001^b^	144(44.3)	109(28.7)	0.039^b^
Glucocorticosteroids, n (%)	311(34.3)	482(34.6)	0.012^b^	142(43.7)	161(42.4)	0.988^b^
**Insulin**						
Insulin infusion, n (%)	738 (81)	1042 (75)	0.001^a^	314 (96.6)	332 (87)	0.011^a^
Insulin infusion total dose IU, mean (SD)	45 (39)	45 (44)	0.018 ^a^	88 (53)	60 (47)	0.013^a^
Insulin rate adjustments, mean (SD)	4.1 (3.4)	3.6 (3.1)	< .001^a^	6.8 (4.4)	5.8 (3.8)	< .001^a^
Insulin bolus, n (%)	83 (9.1)	92 (6.6)	0.018^a^	77 (23.7)	90 (23.7)	0.314^a^
Insulin bolus total dose IU, mean (SD)	2.6 (2.7)	2.8 (3.5)	0.620^a^	4.3 (3.7)	5.9 (10.0)	0.517^a^
**Blood glucose**						
Number of glucose measurements, mean (SD)	6.7 (2.5)	5.9 (2.4)	< .001^a^	8.7 (2.7)	7.6 (2.8)	< .001^a^

SD = standard deviation; n = number; IU = international units

^a^ Linear mixed effect models

^b^ Generalized mixed effect models with logit link function

In total 19,962 glucose levels were collected. Estimates of glucose variability are presented per observation day in Tables 3 and 4. In the unadjusted models, delirium was associated with a higher MAG change (β:0.038; 95% CI:0.017–0.061; p = 0.001) and increased daily delta (β:0.325; 95% CI:0.134–0.494; p = 0.001) in patients without diabetes. After adjustments for potential confounders, the association was not maintained in non diabetic patients using the same definitions for glucose variability (MAG change; β adj.:0.021; 95% CI:-0.004–0.043; p = 0.076 and daily delta β adj.:0.100; 95% CI:-0.096–0.282 p = 0.287). Delirium was positively associated with hypoglycemia in diabetic patients (OR adj.: 2.78; 95% CI: 1.71–6.32, p = 0.005), but not in non-diabetic patients (OR adj.: 1.16; 95% CI: 0.58–2.28, p = 0.689). Generalized mixed- effects models with logit link function were not performed for the association between delirium and severe hypoglycemia as the number of glucose levels below 2.2 mmol/l was insufficient.

**Table 3:** The association between delirium and continuous measures of glucose variability presented per day in non-diabetic and diabetic patients.

Measures of glucose variability	Delirious days, n	Non-delirious days, n	Regression coefficient crude	95% CI	p-value	Regression coefficient adj.	95% CI adj.	p-value
**Non-diabetic patients** *	908	1395						
Mean glucose concentration (mmol/l), mean (SD)	7.25 (0.99)	7.25 (1.09)	0.037	-0.067–0.126	0.466	-0.005^a^	-0.116–0.089	0.940
SD glucose concentration (mmol/l), mean (SD)	1.28 (0.77)	1.26 (0.86)	0.049	-0.013–0.105	0.100	0.027^a^	-0.034–0.088	0.374
MAG change (mmol/l/hr), mean (SD)	0.39 (0.27)	0.36 (0.27)	0.038	0.017–0.061	0.001	0.021^a^	-0.004–0.043	0.076
Daily delta (mmol/l), mean (SD)	3.49 (2.32)	3.28 (2.43)	0.325	0.134–0.494	0.001	0.100^a^	-0.096–0.282	0.287
**Diabetic patients** **	325	380						
Mean glucose concentration (mmol/l), mean (SD)	7.86 (1.43)	8.02 (1.63)	-0.177	-0.4428–0.052	0.157	0.066^b^	-0.121–0.288	0.525
SD glucose concentration (mmol/l), mean (SD)	1.94 (1.07)	1.98 (1.18)	0.021	-0.153–0.202	0.794	0.084^b^	-0.075–0.251	0.291
MAG change (mmol/l/hr), mean (SD)	0.61 (0.39)	0.61 (0.41)	0.030	-0.029–0.088	0.305	0.036^b^	-0.026–0.096	0.235
Daily delta (mmol/l), mean (SD)	5.64 (3.15)	5.56 (3.55)	0.280	-0.246–0.763	0.289	0.339^b^	-0.115–0.777	0.146

* Missing values for mean glucose concentration, SD and daily delta; 0 delirious days and 2 non-delirious days. Missing values for MAG change; 39 delirious days and 136 non-delirious days.

** Missing values for mean glucose concentration, SD and daily delta; 0 delirious days and 1 non-delirious days. Missing values for MAG change; 1 delirious days and 15 non-delirious days.

SD = Standard deviation; MAG = Mean absolute glucose change; CI = confidence interval; adj. = adjusted

^a^ Adjusted for age, gender, BMI, confirmed infection, APACHE-IV-score, SOFA-score, presence of severe sepsis or septic shock, total dose of insulin by continuous infusion per day, total dose of insulin by bolus injections if > 0, energy infusion per day, ICU-observation day > 2, use of corticosteroids, ACE-inhibitors, antipsychotic drugs, cyclosporine or tacrolimus, beta-agonists and norepinephrine.

^b^ Adjusted for age, gender, admission type, APACHE-IV-score, SOFA-score, presence of severe sepsis or septic shock, total dose of insulin by continuous infusion per day, total dose of insulin by bolus injections if > 0, energy infusion per day, ICU-observation day > 2, use of ACE-inhibitors, beta-agonists and beta-blockers.

**Table 4:** The risk of hyper- and hypoglycemia during delirious days in non-diabetic and diabetic patients.

Measures of glucose variability	Delirious days, n (%)Patients, n (%)	Non-delirious days, n (%)Patients, n (%)	OR crude	95% CI	p-value	OR adj.	95% CI adj.	p-value
**Patients without diabetes**	908 (100.0) 323 (100.0)	1395 (100.0) 323 (100.0)						
Hyperglycemia > 8 mmol/l, n (%)	619 (68.2) 251 (77.7)	885 (63.4) 271 (83.9)	1.10	0.96–1.25	0.177	1.04^a^	0.90–1.19	0.594
Severe hyperglycemia > 11 mmol/l, n (%)	146 (16.1) 79 (24.5)	217 (15.6) 79 (24.5)	1.09	0.80–1.45	0.59	0.93^a^	0.66–1.29	0.648
Hypoglycemia < 3.5 mmol/l, n (%)	41 (4.5) 27 (8.4)	39 (2.8) 25 (7.7)	1.45	0.75–2.79	0.243	1.16^b^	0.58–2.28	0.689
Severe hypoglycemia < 2.2 mmol/l, n (%)	6 (0.7) 4 (1.2)	4 (0.3) 4 (1.2)	N.A.	N.A.	N.A.	N.A.	N.A.	N.A.
**Patients with diabetes**	325 (100.0) 87 (100.0)	380 (100.0) 87 (100.0)						
Hyperglycemia > 8 mmol/l, n (%)	278 (85.5) 85 (97.7)	313 (82.4) 84 (96.6)	0.90	0.76–1.07	0.269	0.96^c^	0.81–1.16	0.648
Severe hyperglycemia > 11 mmol/l, n (%)	143 (44.0) 52 (59.8)	173 (45.5) 68 (78.2)	0.88	0.63–1.21	0.451	1.00^c^	0.69–1.33	0.986
Hypoglycemia < 3.5 mmol/l, n (%)	38 (11.7) 29 (33.3)	26 (6.8) 14 (16.1)	2.33	1.24–5.43	0.038	2.78^d^	1.71–6.32	0.005
Severe hypoglycemia < 2.2 mmol/l, n (%)	4 (1.2) 3 (3.4)	4 (1.1) 2 (2.3)	N.A.	N.A.	N.A.	N.A.	N.A.	N.A.

n = number; OR = odds ratio; CI = confidence interval; adj. = adjusted

^a^ Adjusted for age, gender, BMI, confirmed infection, APACHE-IV-score, SOFA-score, presence of severe sepsis or septic shock, total dose of insulin by continuous infusion + bolus injections in 30-minutes before glucose measurement, energy infusion in 30-minutes before glucose measurement, ICU observation day, use of corticosteroids, ACE-inhibitors, antipsychotic drugs, cyclosporine or tacrolimus, beta-agonists and norepinephrine.

^b^ Adjusted for age, gender, BMI, confirmed infection, SOFA-score, presence of severe sepsis or septic shock, total dose of insulin by continuous infusion + bolus injections in 30-minutes before glucose measurement, energy infusion in 30-minutes before glucose measurement, ICU observation day, use of corticosteroids and antipsychotic drugs.

^c^ Adjusted for age, gender, admission type, SOFA-score, APACHE-IV-score, presence of severe sepsis or septic shock, total dose of insulin by continuous infusion + bolus injections in 30-minutes before glucose measurement, energy infusion in 30-minutes before glucose measurement, ICU observation day, use of ACE-inhibitors, beta-agonists and beta-blockers.

^d^ Adjusted for age, gender, admission type, SOFA-score, APACHE-IV-score, total dose of insulin by continuous infusion + bolus injections in 30-minutes before glucose measurement, energy infusion in 30-minutes before glucose measurement, use of beta-agonists and beta-blockers.

We found similar results for glucose variability when all delirious and non-delirious days during ICU stay were analysed compared to the observation days of the first episode, or when consecutive episodes (delirious and non-delirious episodes) were analysed (data not shown).

## Discussion

In this cohort of ICU patients, mean and SD of glucose concentrations, MAG change, daily delta and the risk of hyperglycemia were unaltered during delirious days compared to non-delirious days in non-diabetic and diabetic patients. Furthermore, we demonstrate that in diabetic patients delirium was associated with hypoglycemia. The association was even stronger after adjustment for several confounding factors. This association was not found for non-diabetic patients.

Little is published about the mutual relationship between glucose levels and delirium. It has been reported that mean glucose levels did not differ between patients with delirium and without delirium within non-critically ill older patients [34]. Although we conducted our study in an ICU cohort with critically ill patients, our results are in concordance with their study. In the ICU setting, one study has been conducted reporting higher mean glucose levels in patients with hyperactive delirium compared to patients with non-hyperactive delirium [10]. In our study, we were not able to identify any hyperactive delirium. This may be related to the use of sedatives [24]. Additionally, our study was designed to compare mean glucose concentrations during delirious and non-delirious days per individual. In concordance with our results, tight glucose control has been linked to increased hypoglycaemia rates and increased delirium rates [35, 36]. (Insulin- induced) hypoglycaemia affect brain function [36]. One of the strengths of our study is that we were able to conduct our study in one of the largest high quality cohorts with ICU patients with different delirium episodes [24]. In addition, we had extensive information on glucose measurements, allowing us to assess subtle and detailed changes in glucose levels over time, both in diabetic and non-diabetic patients. In particular, this detailed information enabled us to look at various definitions of glucose variability. Furthermore, we were able to look at within-patient patterns (comparing delirious and non-delirious days in each individual), which minimizes the risk of confounding. Finally, we were able to control for various potential confounders in a time dependent manner, such as glucose-influencing drugs including insulin, norepinephrine, corticosteroids and energy infusion.

However, this study has some limitations. The generalizability is possibly limited as this study was performed as monocenter study at a university hospital. Selection bias could have occurred because patients and observation days without glucose measurements were excluded. Despite our rich information on glucose levels, a potential limitation is the possibility that peaks and nadirs in blood glucose levels have been missed as glucose levels were not measured continuously. We considered this misclassification as non-differential as this misclassification occurred at random during delirious and non-delirious days. Due to the multiple testing, it remains a possibility that the association between delirium and hypoglycemia was based on a type I error, despite the stronger positive association after adjustment for confounders. Unmeasured confounding may have occurred as there could have been unmeasured confounding covariates.

The measures of glucose variability could depend on the number of glucose determinations. Especially, the MAG-change is sensitive for higher frequency of measurement. We consider this as less important because observation days were compared, but not whole ICU stays. Furthermore, we adjusted for disease severity and insulin infusion which indirectly correct for the frequency of measurement. For the number of glucose measurements has not been adjusted because this indices can been seen as glucose variability measure.

Hypoglycemia at the ICU has been associated with increased mortality independent of diabetic status [37]. For this reason, our findings suggest that in clinical practice blood glucose levels should be monitored more often during delirium in critically ill patients with diabetes to avoid hypoglycemia. More research is needed to explore the impact of our findings concerning diabetic patients on ICU outcome and determine whether any causality consists between delirium and glucose variability.

## Conclusions

Mean glucose concentration, its SD, MAG change, daily delta and the risk of hyperglycemia were not significantly altered during delirium in non-diabetic and diabetic ICU patients. Delirium in critically ill patients with diabetes was associated with hypoglycemia. This association was not found for non-diabetic ICU patients. Our findings suggest that glucose levels should be monitored more closely in diabetic patients during delirium at the ICU to prevent hypoglycemia.

## Supporting information

S1 Table Flowchart glucose regulation. (DOC)
