# Supplementary material for: Glucose variability during delirium in diabetic and non-diabetic intensive care unit patients: A prospective cohort study
Source: PLoS One. 2018 Nov 15;13(11):e0205637. doi: 10.1371/journal.pone.0205637 (PMC6237332; doi:10.1371/journal.pone.0205637)
Supplement: S1 Table — (DOC) [file pone.0205637.s001.doc]

**Supporting information**

**S1 table. flowchart glucose regulation**

| **Glucose concentration (mmol/l)** | **Action** | **Check glucose concentration** |
| --- | --- | --- |
| > 20 | Bolus of 8 IU insulin and start or increase dose insulin infusion with 4 IU/hr | 1 hr |
| 16-20 | Bolus of 4 IU insulin and start or increase dose insulin infusion with 2 IU/hr | 1 hr |
| 12-16 | Bolus of 2 IU insulin and start or increase dose insulin infusion with 2 IU/hr | 1 hr |
| 10-12 | Start or increase insulin infusion with 1 IU/hr | 1 hr |
| 8-10 | Decrease of glucose concentration ≥ 50%: decrease dose insulin infusion with 50%  Decrease of glucose concentration < 50%: start or increase dose insulin infusion with 1 IU/hr | 2 hr  3 hr |
| 5-8 | Decrease of glucose concentration ≥ 50%: stop insulin infusion  Decrease of glucose concentration 25-50%: do not change dose insulin infusion  Decrease of glucose concentration < 25%: do not change dose insulin infusion | 1 hr  1 hr  4 hr |
| 3.5-5 | Decrease of glucose concentration ≥ 50%: stop insulin infusion  Decrease of glucose concentration < 50%: decrease dose insulin infusion with 50% | 1 hr  1 hr |
| < 3.5 | Stop insulin infusion and bolus of 25 ml dextrose 50%  (If blood glucose after 0.5 hr > 5.0 mmol/l: start dose insulin infusion after consultation of an intensivist, but increase the insulin infusion in steps of 50%) | 0.5 hr |
| Total Parenteral Nutrition stop | Stop insulin infusion  (If blood glucose after 1 hr > 5.0 mmol/l: start dose insulin infusion with 50% of last dose) | 1 hr |

IU= international units
